# Immunomagnetic Separation Improves the Detection of Mycobacteria by Paper-Based Lateral and Vertical Flow Immunochromatographic Assays

**DOI:** 10.3390/s21185992

**Published:** 2021-09-07

**Authors:** Alejandra Ben Aissa, Barbara Araújo, Esther Julián, Maria Valnice Boldrin Zanoni, María Isabel Pividori

**Affiliations:** 1 Departament de Química, Universitat Autònoma de Barcelona, 08193 Bellaterra, Spain; alejandra.benaissa@eurecat.org (A.B.A.); babiiii.cas@gmail.com (B.A.); 2 Department of Analytical Chemistry, Institute of Chemistry, São Paulo State University, Araraquara 14800-060, SP, Brazil; maria.valnice@unesp.br; 3 Departament de Genètica i de Microbiologia, Facultat de Biociències, Universitat Autònoma de Barcelona, 08193 Bellaterra, Spain; Esther.Julian@uab.cat; 4 Institute of Biotechnology and Biomedicine, Universitat Autònoma de Barcelona, 08193 Bellaterra, Spain

**Keywords:** rapid diagnostic tests, lateral flow assay, vertical flow assay, gold nanoparticles, mycobacteria, double-tagging PCR

## Abstract

This work addresses a method that combines immunomagnetic separation (IMS) and paper-based nucleic acid immunochromatographic assay for the sensitive detection of *Mycolicibacterium fortuitum (basonym Mycobacterium fortuitum)* In particular, the preconcentration of the bacteria was achieved by using magnetic particles modified with an antibody specific towards mycobacteria. Following the IMS, the bacteria were lysed, and the genome was amplified by double-tagging PCR, using a set of primers specific for the 16S rRNA gene for *Mycobacterium*. During the amplification, the amplicons were labeled with biotin and digoxigenin tags. Moreover, a comparative study of paper-based immunochromatographic platforms, relying on vertical and lateral flow and on the use of streptavidin gold nanoparticles as a signal generating system, was also performed. The visual readout was achieved when the gold-modified amplicons were captured by the anti-DIG antibody in the test line. The analytical performance of both methods, nucleic acid vertical flow (NAVF) and nucleic acid lateral flow (NALF), is also discussed. Although NALF showed lower limit of detections (LODs), both NALF and NAVF combined with IMS were able to detect the required LOD in hemodialysis water, becoming two promising and useful techniques for the rapid screening of water supplies in hemodialysis centers, to prevent the exposure of immunosuppressed patients to contaminated sources.

## 1. Introduction

The recent outbreak of a communicable disease that spread globally throughout international borders in a matter of days highlighted the need for rapid diagnostic tests (RDTs), able to provide prompt diagnosis and to help implement security measures to prevent interruption of the transmission chain. Strong research efforts are thus focused on introducing RDTs in low resource settings for the rapid screening of patients requiring treatment, instead of standard clinical diagnostic performed by highly qualified personnel in centralized laboratories [1,2,3,4,5]. Furthermore, because of these rapid tests, there have been increased attention on different areas related to human and animal health [6], as well as other fields, such as food safety, and water and soil quality monitoring [7].

Rapidity, sensitivity, and specificity are not the only features that define these tests, they are also known for their simplicity, robustness, portability and, particularly, low cost [8]. These requirements are compatible with the use of paper as a substrate [9]. Different types of rapid tests are based on cellulose, such as substrate, including immunochromatography [10]. These devices are built by overlapping a series of pads, which enable the flow of the sample and reagents due to capillary forces. In lateral flow formats, the movement of the fluids is produced through the strip by capillarity [7]. Similarly, vertical flow assays are based on the pass of the sample through a series of layered membranes by capillarity, but in this instance, gravity also plays an important role in speeding-up the process [11].

Even though immunochromatographic tests are traditionally used for qualitative and semi-quantitative analysis [12], these devices are also connected to readout systems, such as smart phones, for quantitative applications [12,13]. However, some improvements, in terms of sensitivity, are still needed, as recently highlighted in the case of some antigen LFAs for SARS-CoV-2, attributed to a sampling procedure [14]. In this regard, the preconcentration of the sample plays an important role in improving the limits of detection and sensitivity of the technique. The magnetic particles can be an approach to solve this drawback. The use of these materials, modified with biomolecules, allows for specific biorecognition, followed by the specific capture, magnetic isolation, and preconcentration of a huge range of analytes, including cells, proteins, and small molecules, among others, from a large volume of a complex matrix [15,16,17]. For instance, an electrochemical magneto immunosensor, based on magnetic nanoparticles able to detect foodborne pathogens as low as 1 CFU in 25 mL of milk after 8 h of pre-enrichment, was previously reported on by our research group [18,19].

Moreover, to achieve the required limits of detection, amplification of DNA remains an ineluctable procedure for the sensitive detection of some pathogens. During this pandemic, PCR tests proved to be state-of-the-art and the dominant diagnostic tests for communicable diseases [20]. Unfortunately, the first generation of nucleic-acid amplification technologies based on PCR cannot feasibly be incorporated into “classical” RDTs, since it requires thermocycling platforms, trained personnel, and infrastructure, including a reliable power supply, which constitutes a critical barrier for PCR in scarce-resource settings [21]. Nowadays, there are different solutions to bridge these barriers. For instance, different companies offer cheap, portable, battery-operated thermocyclers [22].

Our group was pioneer in the integration of PCR in RDTs based on electrochemical genosensing devices [23]. Since this early report, different routes, based on PCR, for the amplification of the analytical signal, to increase the sensitivity of the readout, were explored. A double-tagging PCR amplification strategy with electrochemical readout was reported on [24,25]. In this approach, a double-tagged set of primers labeled with small tags (e.g., biotin, digoxigenin, fluorescein, -SH, among others) was used during amplification to achieve immobilization on the platform and further readout. This method can easily be multiplexed, up to three pathogens [26]. Even improved analytical features were achieved by combining double-tagging amplification with immunomagnetic separation (IMS) [19]. The integration of double-tagged amplicon to RDTs based on immunochromatography was also reported [27,28], showing a comparable sensitivity for the detection of double-tagged amplicons: electrochemical biosensing and lateral flow labeled with Au-NPs [28] and carbon nanoparticles [29].

In this paper, the integration of immunomagnetic separation, the double-tagging PCR amplification, and two different immunochromatography paper-based platforms are presented, for the detection of *Mycobacterium fortuitum*. This is one of the most common mycobacteria in contaminated hemodialysis waters, which can put immunocompromised patients at risk. Hence, it is important to continuously monitor water supplies in hemodialysis centers, to minimize the exposure of renal transplantation and immunocompromised individuals to contaminated sources [30]. In detail, this work presents a comparative study of the two paper-based platform configurations, based on nucleic acid immunochromatography. The role of the IMS performed with anti-Mycobacteria antibody functionalized magnetic particles was also studied [31].

## 2. Materials and Methods

### 2.1. Instrumentation and Materials

Glass fiber conjugate pad (GFCP083000) and cellulose fiber sample pad strips (CFSP203000) were purchased from Millipore (Burlington, MA, USA). Absorbent pads (CF7) and nitrocellulose membranes (FP120HP) were from GE Healthcare (Chicago, IL, USA). Whatman^®^ Protran^®^ nitrocellulose membrane used for vertical flow devices (Z670634) was provided by Sigma (Merck KGaA, Darmstadt, Germany). Gentle Tape 2.5 cm × 5 m roll was from Leukopor and Miriad RVF cartridges were acquired from Euromedex (Souffelweyersheim, France). Lateral flow strips Adhesive Backing Cards were obtained from Kenosha C.V. (Amstelveen, The Netherlands). The dispensing of the test and control line was performed using a Lateral Flow Reagent Dispenser from Claremont Bio (Upland, CA, USA) combined with the KDS Legato™ 200 series syringe pump from KD Scientific, Inc. (Holliston, MA, USA).

### 2.2. Chemicals and Biochemical

InnovaCoat^®^ GOLD 40 nm Streptavidin gold nanoparticles (streptAv-AuNPs) were purchased from Innova Biosciences (Cambridge, UK). Dynabeads™ M-280 Tosylactivated magnetic particles (2.8 μm, 30 mg mL^−1^, 2 × 10^9^ MP mL^−1^) were provided by Invitrogen Dynal AS (Oslo, Norway).

Polyclonal antibody to anti-LAM from *Mycobacterium* (MBS315001) was from MyBioSource (San Diego, CA, USA). All buffer solutions were prepared with Milli-Q water and all other reagents were in analytical reagent grade (supplied from Sigma). The composition of these solutions were: borate buffer (0.1 mol L^−1^ boric acid, pH 8.5); conjugate diluting buffer (2 mmol L^−1^ borate pH 7, 10% *w*/*v* sucrose); running buffer (0.01 mol L^−1^ phosphate buffer pH 7.4, 1% BSA, 0.05% Tween 20); ammonium sulfate buffer ((NH_4_)_2_SO_4_ 3 mol L^−1^ in borate buffer); blocking buffer/PBS 0.5% BSA (10 mmol L^−1^ phosphate, 0.5% *w*/*v* BSA, pH 7.4); washing buffer/PBS 0.1% BSA (10 mmol L^−1^ phosphate, 0.1% *w*/*v* BSA, pH 7.4); sample pad buffer (0.01 mol L^−1^ phosphate buffer pH 7.4, 1% BSA, 0.05% Tween 20).

### 2.3. Oligonucleotides Sequences

The oligonucleotides were obtained from Sigma (Berlin, Germany). These sequences, complementary to the region of 16S rRNA gene common to all members of the genus *Mycobacterium*, were selected from a previous publication [32].

Biotin (BIO) and digoxigenin (DIG) were used to achieve the double tagging of the amplicon, both inserted in 5′ end of the primers. The primer sequences as well as the tags used for the PCR amplification are shown in Table 1.

### 2.4. Preparation of the Devices in Vertical Flow Configuration

**Figure 1:**
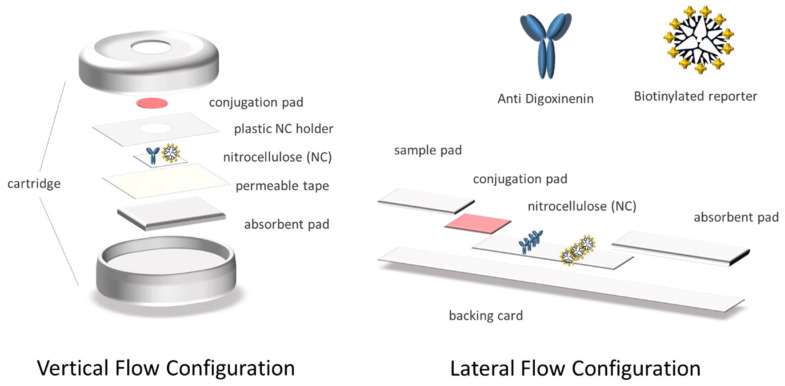
Schematic representation of the components forming the vertical and lateral flow configuration.

The different pads conforming the system were assembled and placed in the cartridge, as shown in Figure 1. Afterward, 0.5 μL of the anti-digoxigenin antibody (1 mg mL^−1^) and the positive control biotinylated reporter [27] (1 mg mL^−1^) were deposited on the nitrocellulose membrane and were then dried at RT for 30 min. Furthermore, the streptAv-AuNPs were diluted 5 times in conjugate diluting buffer, embedded in the glass fiber conjugate pads, and then dried for 3 h at RT. Finally, the glass fiber was placed on the corresponding well of the device, in direct contact with the test and control area of the nitrocellulose.

### 2.5. Preparation of Devices in Lateral Flow Configuration

The cellulose fiber sample pads were soaked into the sample pad buffer. The streptAv-AuNPs were then diluted 10 times in conjugate diluting buffer and embedded in the glass fiber conjugate pads. All of the pads were dried for 3 h at RT. The antibody (1 mg mL^−1^) and the positive control biotinylated reporter (1 mg mL^−1^) [27] were dispensed on the nitrocellulose membrane and dried at RT for 1h. Finally, the strips were assembled on the adhesive backing card as it is shown in Figure 1.

### 2.6. Covalent Immobilization of Antibodies on Magnetic Particles

**Figure 2:**
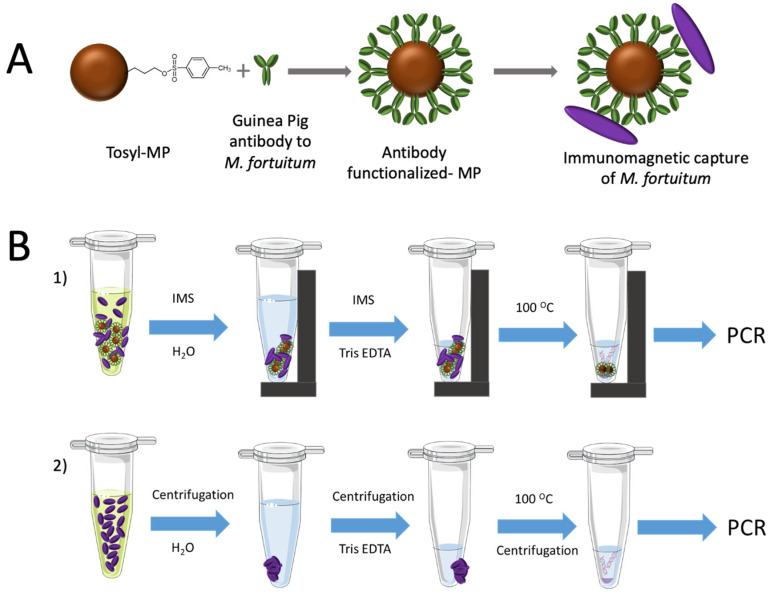
Panel (**A**) covalent immobilization of the antibody specific to *x* on Tosyl-MP. Panel (**B1**) schematic procedure for the DNA extraction performed after immunomagnetic separation and magnetic preconcentration of *M. fortuitum*. Panel (**B2**) schematic procedure for the DNA extraction performed from culture media dilutions.

The antibody to *M. fortuitum* was covalently coupled on tosyl activated magnetic particles (tosyl-MPs) as described in Figure 2, Panel A.

A volume of 35 μL of tosyl-MP was washed twice with 1 mL of borate buffer. Afterwards, 20 μg of antibody and 100 μL of ammonium sulfate buffer was added in borate buffer performing a total volume of 240 μL.

MPs were incubated under continuous agitation for a total reaction time of 18 h at 37 °C. After incubation, MPs were separated with a magnet and the supernatant was removed. Afterward, 1 mL of phosphate blocking buffer was added to the suspension and incubated under shaking for 2 h at 37 °C, in order to block the remaining tosyl groups of the magnetic particles.

Finally, the antibody functionalized-magnetic microparticles were washed and resuspended in phosphate storage buffer to reach a concentration of 5.3 mg mL^−1^ and were then stored at 4 °C for further use for 1 month, as recommended by the manufacturer.

### 2.7. Bacterial Strain and Culture

*Mycolicibacterium fortuitum* subsp. *fortuitum* ATCC^®^ 6841™ strain was grown in trypticase soy broth (TSB) supplemented with 0.025% Tween 20 under continuous agitation at 37 °C for 72 h. Afterward, serial dilutions from the culture were performed in hemodialysis water. Hemodialysis water was used since non-tuberculous mycobacteria are associated with infection, arising from contamination of water in hemodialysis patients. The hemodialysis water was kindly provided by the Servei de Nefrologia, Hospital Parc Taulí Sabadell. A total of 100 μL of each dilution was spread onto Tryptic Soy Agar plates. After incubating the plates at 37 °C for 24 h, the culture colonies on the plates were counted to estimate the number of viable bacteria in Colony Forming Units (CFU mL^−1^).

### 2.8. Immunomagnetic Separation and DNA Extraction

The magnetic separation of the mycobacteria was performed to preconcentrate the bacteria from high volume of sample under magnetic actuation, and in order to improve the limit of detection (LODs). To achieve that, 20 μL of the antibody functionalized-magnetic particles was added to 1 mL of each mycobacteria samples (ranging from 0 to 10^6^ CFU mL^−1^) and were incubated by stirring at RT for 60 min.

Afterwards, the bacteria attached to the MPs were separated with a magnet and the supernatant was removed, as depicted in Figure 2, Panel B1. Then, MPs were washed with PBST (X3) and water (X1) under shaking for 1 min at RT and 700 rpm. The particles were separated with a magnet and resuspended in 50 μL of Tris-EDTA (TE) buffer for DNA extraction. The suspension was then kept in a boiling water bath for 10 min. After cooling on ice for 5 min, 2 μL of the supernatant was used directly for the amplification.

In order to assess the efficiency of the preconcentration with magnetic particles, a parallel extraction of DNA from the same solutions was performed with no magnetic preconcentration, as shown in Figure 2, Panel B2. In this case, 1 mL of each one of the dilutions of mycobacteria were centrifuged at 12,000× *g* for 15 min, the liquid cultures were removed, and the pellet was resuspended in 1 mL of water. After a second centrifugation at 12,000× *g* for 15 min, the suspension was kept in a boiling water bath for 10 min. After cooling on ice for 5 min, the samples were centrifuged and 2 μL of the supernatant was used for the amplification samples.

### 2.9. Double-Tagging PCR for the Amplification of Mycobacteria

**Figure 3:**
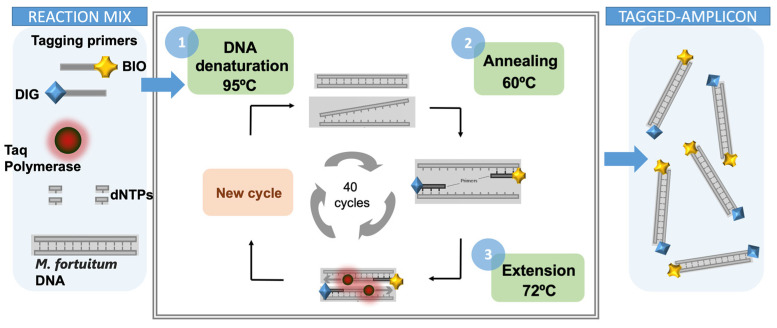
Schematic representation of the double-tagging PCR amplification, in order to obtain the double-tagged amplicon labeled with biotin (BIO) and digoxigenin (DIG) from *M. fortuitum* DNA.

A set of primers tagged with biotin (BIO) and digoxigenin (DIG), respectively, was used for the amplification and the double tagging of the extracted DNA. The PCR was performed in 15 μL of reaction mixture containing 2 μL of the extracted DNA. Each reaction contained 250 μmol L^−1^ of each deoxynucleotide triphosphate (dATP, dGTP, dCTP, and dTTP), 100 nmol L^−1^ of the double-tagged set of primers, and 3 U of Taq polymerase. The reaction was carried out in Taq DNA Polymerase PCR 1x Buffer (BioTools) containing 7.5 mM Tris HCl (pH 9.0), 0.2 mM MgCl_2_, 5 mM KCl, 2 mM (NH_4_)_2_SO_4_. The amplification mixtures were treated, with an initial step at 95 °C for 5 min followed by 40 cycles at 95 °C for 40 s, 6 °C for 30 s, and 72 °C for 2 min, and a last step of 10 min at 72 °C. The resulting samples were stored at 4 °C. The detailed PCR programing conditions for the double-tagging PCR are presented in Table S1 in the Supplementary Material section, and schematically shown in Figure 3.

The performance of the multiplex double-tagging PCR was analyzed with conventional agarose gel electrophoresis on 2% agarose gel in TAE buffer containing 1 × GelRed dye. A molecular weight (MW) marker consisting of DNA fragments ranged from 100 to 1000 base pair (bp) was used as size amplicon control. The DNA bands were visualized by UV transillumination (FastGene FAST Digital System).

### 2.10. Nucleic Acid Vertical Flow

**Figure 4:**
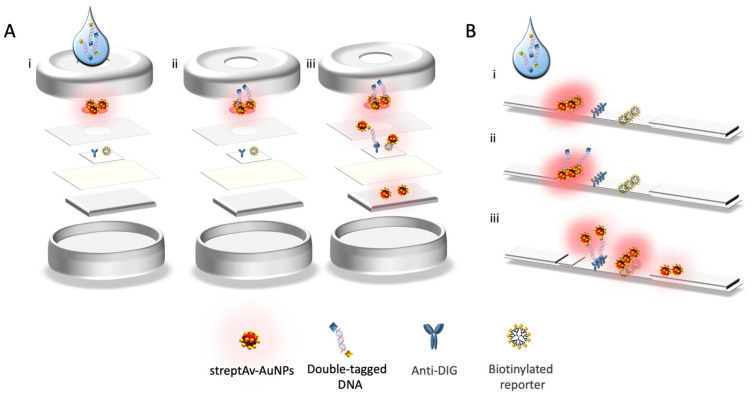
Schematic representation of Nucleic Acid Vertical Flow (**A**) and Nucleic Acid Lateral Flow (**B**): (i) deposition of the amplicons in the sample area; (ii) streptavidin gold nanoparticles (streptAv-AuNPs) captured by the biotin tag of the amplicon; (iii) streptAv-AuNPs Amplicon capture in the test area (dot or line, respectively) by the anti-DIG antibody and capture of gold nanoparticles by the biotinylated reporter [27]. The remaining streptAv-AuNPs flow up to the absorbent pad.

The procedure for the detection of mycobacteria by Nucleic Acid Vertical Flow (NAVF) is schematically described in Figure 4, Panel A. The amplicon solutions obtained from the double-tagging PCR were diluted in 75 μL of running buffer and deposited on the sample zone. After 20 s, 75 μL of running buffer were added in order to drag the remaining streptAv-AuNPs to the absorbent pad. The streptAv-AuNPs thus reacted with the biotin (BIO-tag) of the amplicons. As the products moved towards the nitrocellulose, the streptAv-AuNPs/amplicons were captured by the digoxigenin (DIG-tag) on the test dot, containing the antibody anti-DIG. A valid test was considered when the streptAv-AuNPs also reacted with a biotinylated reporter located on the control dot. The reporter is a biotinylated reporter previously described by our research group [27], although good performance can be achieved by using other biotinylated proteins at higher concentration levels [29].

After one minute, the remaining streptAv-AuNPs migrated to the absorbent pad and the visual readout was achieved.

### 2.11. Nucleic acid Lateral Flow

The procedure for the detection of mycobacteria by Nucleic Acid Lateral Flow (NALF) is schematically described in Figure 4, Panel B. The amplicon solutions obtained from the double-tagging PCR amplifications was diluted in 100 μL of PBS running buffer and deposited on the sample zone. After 1 min, 100 μL of running buffer was added in order to transport the remaining streptAv-AuNPs up to the absorbent pad. Similarly, to the vertical flow immunoassay, the streptAv-AuNPs reacted with the biotin of the amplicons (BIO-tag). When streptAv-AuNPs/amplicon moved through the nitrocellulose, the specific antibody anti-DIG on the test line reacted with the DIG-tag of the amplicons. The remaining streptAv-AuNPs flowed through the nitrocellulose up to the control line where they were captured by the biotinylated reporter [27] used as a positive control. After 10 min, the test revealed the lines, and the test was ready for the interpretation of the results.

### 2.12. Data Interpretation and Analysis

In order to avoid any bias in the images due to luminosity, all of the strips for a single piece of experiment were photographed in a single image, and the conditions were the same in all cases. The images were taken, at the same time, for all of the strips in a single image, using a portable photographic studio based on LED lights of 1100 lm and a color temperature of 6000–6500 K. The images were taken at a distance of 23 cm with a smartphone. The rear camera was used, with a maximum resolution of 12 megapixels (4032 × 3024 pixels). Autofocus was enabled, and the flashlight was turned off during the data acquisition procedure. Then, the images were converted to an 8-bit grey-scale format using the command Image > Type > 8-bit. The test and control lines were outlined using the rectangular selection tool, and the area under each peak was then numerically integrated using the ImageJ gel analysis toolbox.

## 3. Results and Discussion

### 3.1. Double-Tagging PCR for the Amplification of Mycobacteria

**Figure 5:**
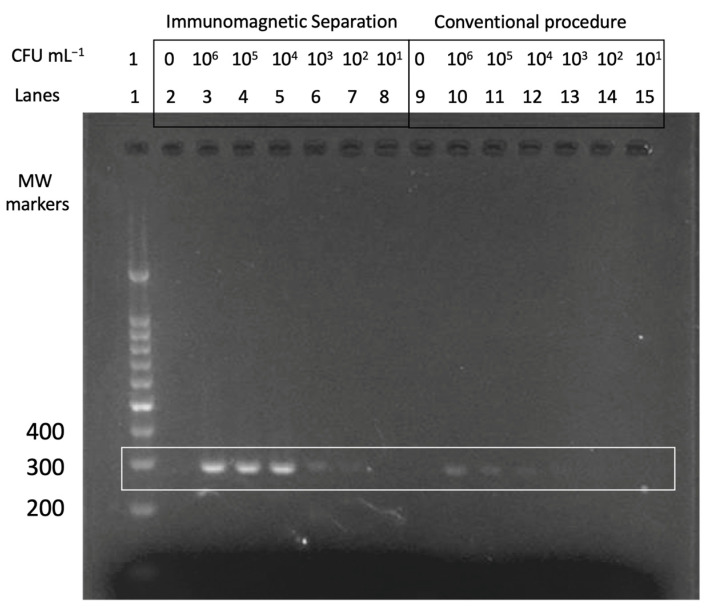
Gel electrophoresis. Lane 1 is the molecular weight (MW) marker. Lanes 2 to 8 were obtained by the double tagging PCR combined with IMS. Lane 2 is the negative control of this procedure and corresponds to 0 CFU mL^−1^. Lanes 3 to 8 range from 10^6^ to 10^1^ CFU mL^−1^. Lanes 9 to 15 are the samples resulting from the double tagging PCR obtained by conventional DNA extraction, instead of IMS, from 10^6^ to 10^1^ CFU mL^−1^ (lanes 10 to 15). Lane 9 is the negative control of this procedure and correspond to 0 CFU mL^−1^.

The double-tagging PCR procedure (schematically depicted in Figure 3) was firstly studied by end-point PCR and agarose gel electrophoresis; the results are shown in Figure 5. The resulting bands showed an molecular weight (MW), as expected, for the 16S fragment (277 bp).

From the results, it can be observed that the band intensities resulting from the samples obtained by immunomagnetic separation (lanes 2 to 8) are much higher than the conventional procedure involving centrifugation (lanes 10 to 15). An expected decrease in the signal was also observed when lowering the concentration of the bacteria.

### 3.2. Nucleic Acid Vertical Flow

**Figure 6:**
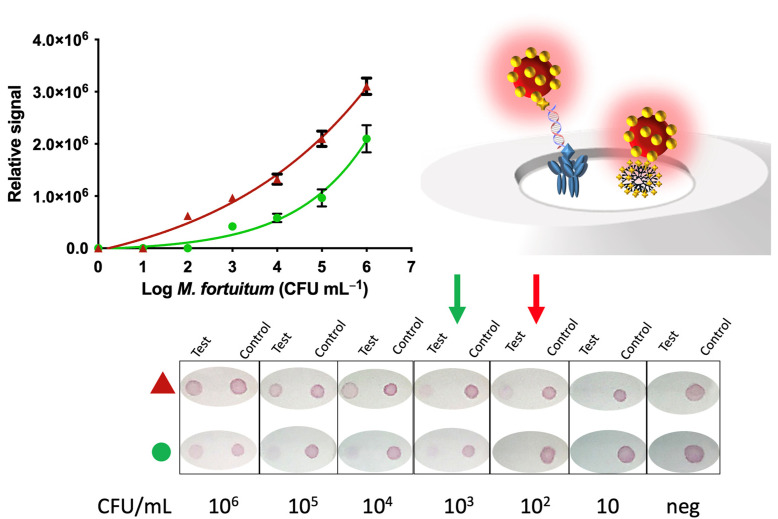
Results obtained for the NAVF for different CFUs mL^−1^ of *M. fortuitum* submitted to immunomagnetic separation and preconcentration by magnetic actuation (▲) and with no magnetic preconcentration (●). The negative control for the evaluation of non-specific binding is also shown.

Figure 6 shows the results obtained by NAVF. Regarding the performance of the device, it is important to highlight that the readout can be achieved in only 1 min. The results of the tests can be read with the naked eye, but the measurement of the intensity of the dots using the image-processing program ImageJ allows the semi-quantification and an enhanced evaluation of the data. After processing the images resulting from the addition of the amplicons to the detection area of the devices, the intensity of color produced for each concentration of mycobacteria was quantified, and the results are shown in Figure 6. Figure S1 (Supplementary Material) shows the relative areas for each type of sample pretreatment, after processing the images. These areas were used for plotting Figure 6. In all instances, the negative control containing all reagents, except the DNA template, was also processed, showing no visual signal, and confirming negligible non-specific adsorption of the signal generating system. Moreover, a clean white area around the reaction dots is observed in all cases, showing only a specific reaction in the reaction dot and negligible non-specific adsorption on the bare membrane, as confirmed in the bare areas showed in Figure S1 (Supplementary Material).

The relative areas (Figure S1, Supplementary Material) were then fitted using a nonlinear regression (four parameter logistic equation—GraphPad Prism Software) for the samples submitted to immunomagnetic separation and preconcentration by magnetic actuation (R^2^ = 0.9881, red plot) and for the samples not submitted to magnetic actuation (R^2^ = 0.9620, green plot), for n = 3 in any case. The IMS/NAVF method was able to visually detect signals as low as 100 CFU mL^−1^ (red arrow, Figure 6). This strategy showed better results than those obtained from the method without IMS obtaining a result of 10^3^ CFU mL^−1^ (green arrow, Figure 6). This difference can be attributed to a lower performance of the sample treatment obtained by the centrifugation process (depicted in Figure 2, Panel B2) compared to IMS (depicted in Figure 2, Panel B1). IMS provided high affinity and specificity of the immobilized antibody. Therefore, the IMS step followed by the magnetic preconcentration improved, by an order of magnitude, the LOD for the detection of *M. fortuitum* in hemodialysis water detected by NAVF.

### 3.3. Nucleic Acid Lateral Flow Immunoassay

The results combining double-tagging PCR and lateral flow immunoassay integrating the IMS of the mycobacteria (or alternatively, the centrifugation) were quantified as previously described for NAVF. The readouts in this instance were obtained after 15 from the addition of the sample on the strip.

**Figure 7:**
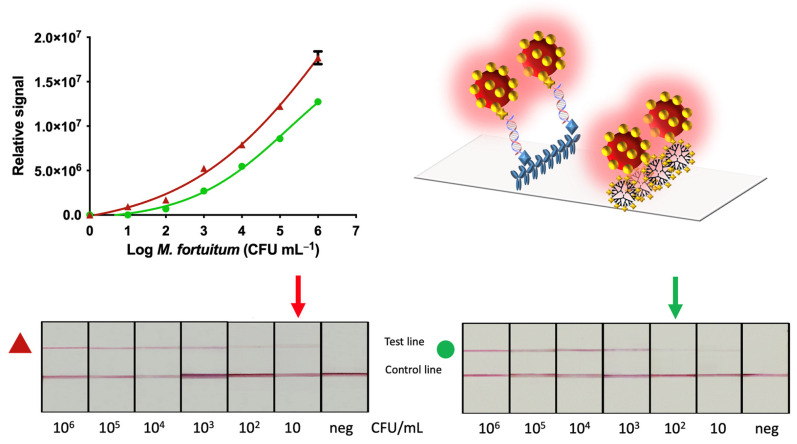
Results obtained for the NALF for different CFU of *M. fortuitum* submitted to immunomagnetic separation and preconcentration by magnetic actuation (▲) and with no magnetic preconcentration (●). The negative control for the evaluation of non-specific binding is also shown.

Figure S1 (Supplementary Material) shows the relative areas for each type of sample pretreatment, after processing the images. These areas were used for plotting Figure 7.

The results for both approaches, IMS/NALF compared to the common procedure based on centrifugation are shown in Figure 7. In this case, the relative areas (showed in Figure S1, supplementary data) were fitted using a nonlinear regression (four parameter logistic equation—GraphPad Prism Software) (R^2^ = 0.9949 for IMS/NALF and 0.9445 for NALF).

The LOD obtained from the procedure combining IMS was 10 CFU mL^−1^ (red arrow, Figure 7), 10 times lower than the value obtained from samples processed by centrifugation (10^2^ CFU mL^−1^, green arrow, Figure 7), being the results in agreement with those obtained by NAVF.

Furthermore, the comparison of the analytical performance of NAVF compared to NALF showed an improved result for NALF in one order of magnitude. The improved LOD of NALF can be attributed to the fact that, due to the configuration of the assay, all of the gold nanoparticles, as well as the sample, were forced to pass by the test and control lines, ensuring increased contact of the reagents and providing much more opportunities for reaction. Unlikely, although much more rapid, since the flow is also moved by gravity in the vertical flow format, some reagents (including gold nanoparticles and samples) can bypass the membrane without reacting with the control and test dots (as depicted in Figure 4). 

For this reason, it can be concluded that, to ensure detection at high sensitivity, the lateral flow would be the best candidate to achieve improved results in terms of LOD. However, the time of detection and the quantity of materials required for NAVF makes this format very attractive for applications that require qualitative results, ensuring, in any case the correct performance to reach the proper LOD for each necessity.

## 4. Conclusions

There has been increasing interest in commercial solutions and rapid diagnostic testing developments to meet the need of analyzing targets in complex samples, at low concentration levels, and in low-resource settings. As there is increasing demand for decentralized infectious disease testing, highlighted by the pandemic, researchers have stepped up in their efforts to provide solutions that meet the WHO guidelines, defined by the acronym ASSURED: (A) affordable, (SS) sensitive, specific, (U) user-friendly, (R) rapid and robust, (E) equipment free, and (D) deliverable to those who need it. In line with this, the integration of technologies with proven performance records in life sciences research, such as immunoassays or PCR amplification being used with smartphones and tablets as readers, has led to cheaper, hand-held devices.

In this work, the performance of immunomagnetic separation of mycobacteria, followed by double-tagging PCR and optical detection based on paper-based immunochromatography are presented in different formats: vertical and lateral flow.

First, the specific separation of the bacteria from the hemodialysis water was performed using magnetic particles modified with an anti-lipoarabinomannan (LAM) antibody. LAM is a glycolipid, known as a virulence factor, associated with mycobacteria. Mycobacterial DNA was then amplified by using a set of primers for the 16S rRNA gene for *Mycobacterium*; the signal was amplified at the same time by labeling with biotin (BIO) and digoxigenin (DIG) to achieve a visual readout by NAVF or NALF, using both gold nanoparticles as the signal generating system.

This approach solves one of the main issues regarding the implementation of LFA for the detection of the whole bacteria cell. Due to the size of the bacteria, pore size must be high, thus increasing the flow rate and compromising the sensitivity. If the flow rate is very fast, the capturing process cannot be complete, and line intensities become unclear [33], compromising the limit of detection. For the bacteria to flow, many authors reported lateral flow assays for bacterial fragments obtained by heating at 100 °C [34]. LOD is quite high (10^5^ in most of the instances) and, thus, only useful for the detection of pure cultures [34]. Our strategy relied on combining immunomagnetic separation and DNA amplification to increase the sensitivity of the approach (up to 10 CFU mL^−1^).

Including immunomagnetic separation provided results, as great as one order of magnitude compared to the same method of analysis without magnetic preconcentration. NALF showed improved LODs that were able to detect as low as 10 CFU mL^−1^ when the IMS was included in the procedure compared to the 100 CFU mL^−1^ when IMS was not included. The LOD of NAVF also improved (100 CFU mL^−1^) when the IMS was performed. The results of NALF are comparable to those obtained in the literature (13 CFU mL^−1^) using magneto-ELISA using conventional benchtop equipment to achieve the readout [31], representing an improvement, in terms of time, with respect to the gold standard methods, which require at least 72 h to detect rapid growing mycobacteria. On the other hand, the time of the assay for the vertical flow format was only 1 min in front of the 15 min needed to obtain the results by using the lateral flow format. Although both platforms have differences, both methods presented in this study were able to detect concentrations of this important pathogen in application, such as dialysate fluid (<200 CFU mL^−1^). This is one of the most “found” mycobacteria in contaminated hemodialysis waters, placing immunocompromised patients at risk. Hence, it is important to continuously monitor water supplies in hemodialysis centers, in order to minimize the exposure of contaminated sources to renal transplantation patients and immunocompromised individuals [32]. Future research will focus on a clinical validation of the method, including the detection of isolations from different clinical samples.

## Figures and Tables

**Table 1:** Sequences of the set of primers for the double-tagging PCR amplification for the detection of *M. fortuitum*.

Strain	Gene	Primer Sequence	Type	5’ Label	Size (bp)
*M. fortuitum*	*16 s*	ACTTGCGCTTCGTCCCTAT	Forward	Biotin	287
		ACCACGCATTTCATGGTCT	Reverse	Digoxigenin	

## Data Availability

Not applicable.

## Supplementary Materials

The following are available online at https://www.mdpi.com/article/10.3390/s21185992/s1, Table S1: Thermal cycler conditions for the double-tagging PCR, Figure S1: The relative areas obtained after processing the images, and which were plotted in Figure 6 and Figure 7.
