# Supplementary material for: Immunomagnetic Separation Improves the Detection of Mycobacteria by Paper-Based Lateral and Vertical Flow Immunochromatographic Assays
Source: Sensors (Basel). 2021 Sep 7;21(18):5992. doi: 10.3390/s21185992 (PMC8468674; doi:10.3390/s21185992)
Supplement: Supplementary file 1 [file sensors-21-05992-s001.zip › sensors-1346822-supplementary.pdf]

sensors MDPI

Supplementary Material

Immunomagnetic separation improves the detection of mycobacteria by paper-based lateral and vertical flow immunochromatographic assays

Alejandra Ben Aissa[1], Barbara Araújo[1,4], Esther Julian[3], Maria Valnice Boldrin Zanoni[4] and María Isabel Pividori [1,2,*]

1 Departament de Química, Universitat Autònoma de Barcelona, 08193, Bellaterra, Spain.
2 Institute of Biotechnology and Biomedicine, Universitat Autònoma de Barcelona, Bellaterra, Spain.
3 Departament de Genética i de Microbiologia, Facultat de Biociències, Universitat Autònoma de Barcelona, Bellaterra, Spain
4 Department of Analytical Chemistry, Institute of Chemistry, UNESP-Univ Estadual Paulista, Araraquara, SP, Brazil

* Correspondence: Isabel.Pividori@uab.cat; Tel.: (+34 93 581 1976)

2. Materials and Methods

2.9. Double-tagging PCR for the amplification of Mycobateria

Table S1. Thermal cycler conditions for the double-tagging PCR

	Initial step	DNA denaturation	Annealing	Extension	Last step
	1 cycle	40 cycles			1 cycle
**Temperature (ºC)**	95	95	60	72	72
**Time (s)**	300	40	30	120	600

3. Results and Discussion

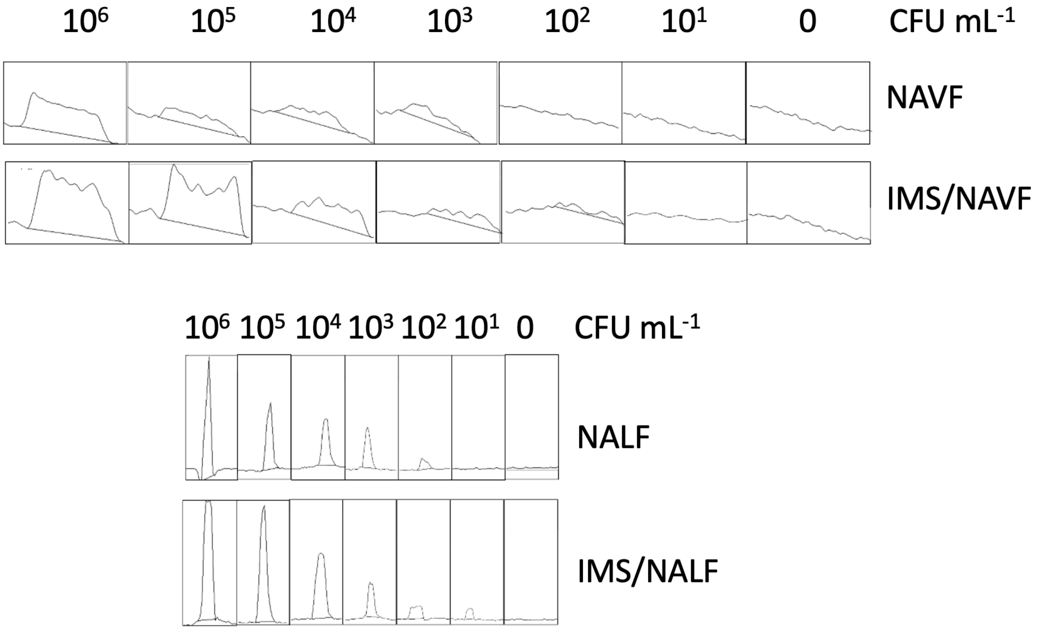

Figure S1. The relative areas obtained after processing the images, and which were plotted in Figure 6 and 7, are shown for both platforms and sample treatments. NAVF, Nucleic Acid Vertical Flow; IMS, immunomagnetic separation; NALF, Nucleic Acid Lateral flow.
